# Functional role of ADAMTS5 in adiposity and metabolic health

**DOI:** 10.1371/journal.pone.0190595

**Published:** 2018-01-02

**Authors:** Dries Bauters, Pierre Bedossa, Henri Roger Lijnen, Bianca Hemmeryckx

**Affiliations:** 1 Center for Molecular and Vascular Biology, Department of Cardiovascular Sciences, University of Leuven, Leuven, Belgium; 2 Department of Pathology, Hôpital Beaujon, Clichy, France; State University of Rio de Janeiro, BRAZIL

## Abstract

Previous studies with gene-deficient mice (ADAMTS5-P) revealed that ADAMTS5 (A Disintegrin And Metalloproteinase with Thrombospondin type 1 motifs, member 5) plays a functional role in adiposity and metabolic health. To confirm these observations, we have performed similar studies with an independently generated strain of ADAMTS5 deficient mice (ADAMTS5-J). Upon cold exposure as well as after high-fat diet feeding (diet-induced obesity or DIO model), these knockout (KO) mice developed less subcutaneous and gonadal white adipose tissue (WAT) as compared to their wild-type (WT) littermates (reduction was more pronounced in ADAMTS5-P mice). Enhanced browning of WAT, as monitored by expression of UCP-1 was seen in the ADAMTS5-J KO mice upon cold exposure but not in the DIO model (seen in both conditions with the ADAMTS5-P mice). Brown adipose tissue (BAT) mass was not different between KO and WT ADAMTS5-J mice, either upon cold exposure or in the DIO model (in contrast to the enhanced BAT mass with the ADAMTS5-P mice). Energy expenditure and thermogenesis were not significantly different between KO and WT ADAMTS5-J mice (in contrast to somewhat enhanced levels in ADAMTS5-P mice). Insulin sensitivity was improved in the ADAMTS5-J KO mice, and they were protected against non-alcoholic steatohepatitis in the DIO model (as the ADAMTS5-P mice). These data are thus similar for both strains of KO mice, confirming specificity of the phenotype, but some quantitative and qualitative differences are also observed.

## Introduction

Increasing energy expenditure by stimulating thermogenesis through activation of brown adipose tissue (BAT) and/or induction of browning of white adipose tissue (WAT) is considered a promising strategy to treat/prevent obesity and related metabolic diseases [1–3]. Whereas WAT is adapted to store energy as triglycerides, BAT produces heat (non-shivering thermogenesis). In brown adipocytes, the uncoupling protein-1 (UCP-1) regulates conversion of energy into heat by uncoupling ATP production from mitochondrial respiration [4]. Also in WAT adaptive UCP-1 positive adipocytes (brown in white: brite or beige) can arise, predominantly in subcutaneous (s) WAT [5]. This browning of WAT is enhanced by exposure to cold and in response to β_3_-adrenergic receptor (β_3_-AR) agonists [6]. It was reported that increasing the mass of brown and beige adipocytes in man indeed resulted in reduction of body weight and fat mass, associated with improved glucose metabolism and insulin sensitivity [7].

Several mechanisms have been identified that contribute to browning of WAT (reviewed in [8, 9]), but a specific causative pathway constituting a clear therapeutic target has not yet been proposed. We have previously identified ADAMTS5, the main aggrecanase [10], as a protein that appears to play a functional role in development of WAT and BAT and in browning of WAT [11, 12]. ADAMTS5 deficient or knockout (*Adamts5*^-/-^ or KO) mice [13] indeed showed lower WAT mass, higher mass of interscapular BAT and enhanced browning of WAT, as indicated by elevated UCP-1 levels. KO mice displayed a small but significant improvement in metabolism and insulin sensitivity as well as enhanced thermogenesis [12, 14]. These findings suggested that ADAMTS5 antigen and/or activity would promote WAT development and impair BAT development or browning of WAT. In addition, *Adamts5*^-/-^ mice appeared to be protected from non-alcoholic steatohepatitis (NASH) when kept on a Western type diet [14]. Thus, neutralization of ADAMTS5 could be a potentially interesting strategy to combat obesity and to improve metabolic health.

To provide confirmation of these observations, we have obtained a second strain of ADAMTS5 deficient mice, generated independently [15]. In the present study, we report that the phenotype of these KO mice shows some similarities with the originally studied strain, but is much less pronounced.

## Materials and methods

### Animals and experimental models

Heterozygous ADAMTS5-P mice [13] were a kind gift from Prof J. Sandy (Rush University Medical Center, Chicago, USA), and heterozygous ADAMTS5-J mice [15] from Prof A. Fosang (Murdoch Childrens Research Institute, University of Melbourne, Australia). The latter are also available from Jacksons Lab (#005771). The ADAMTS5-P mice were originally generated by Pfizer, in collaboration with Lexicon Genetics (The Woodlands, TX, USA) [13, 16]. Briefly, exon 2 was flanked by loxP sites, in combination with Cre-recombinase under control of the EIIa-promoter. Mice were backcrossed with C57Bl/6J mice for several generations to obtain a 99.6% C57Bl/6J genetic background. Alternatively, the ADAMTS5-J mice were generated by Deltagen Inc. [15] and are now commercially available through The Jackson Laboratory (http://www.informatics.jax.org/external/ko/deltagen/1232_MolBio.html, Bar Harbor, USA). The targeting construct, containing an IRES-lacZ-neomycin cassette, substituted 134 nucleotides of exon 2.

ADAMTS5 wild-type (WT or *Adamts5*^+/+^) and homozygous deficient (*Adamts5*^-/-^) littermate mice were generated from heterozygous breeding pairs and genotyped as described [13, 15] (http://dx.doi.org/10.17504/protocols.io.j9wcr7e; http://dx.doi.org/10.17504/protocols.io.kaucsew). All animal experiments were approved by the local Ethical Committee for Animal Experimentation (KU Leuven, P016/2013) and performed in accordance with the NIH Guide for the Care and Use of Laboratory Animals (1996).

For the diet-induced obesity (DIO) model, eight-week-old male mice had *ad libitum* access to drinking water and were kept on standard chow (SFD, 10.9 kJ/g) or on western high-fat diet (HFD; 22 kJ/g; kcal from 42% fat, 43% from carbohydrates and 15% from protein; E15721-34, Ssniff, Soest, Germany) for 15 weeks (http://dx.doi.org/10.17504/protocols.io.kbacsie). The animals were housed in a temperature-controlled room with a 12-hour light/dark cycle. Body weight and food intake were measured at regular intervals.

In separate experiments, 8-week-old, male *Adamts5*^+/+^ and *Adamts5*^-/-^ mice were exposed to cold (4–8°C) for 72 h or 2 weeks in a ventilated cooling unit (Friginox, France) with a 12-hour light/dark cycle (http://dx.doi.org/10.17504/protocols.io.kbbcsin). Mice were housed individually and had *ad libitum* access to standard chow.

At the end of the experiments, mice were fasted for 6 h and sacrificed by *i*.*p*. injection of 60 mg/kg Nembutal (Abbott Laboratories, North Chicago, IL, USA). Blood was collected via the retro-orbital sinus on trisodium citrate (final concentration 0.01 M), and plasma was stored at -80°C. Intra-abdominal gonadal (GON) and sWAT, interscapular BAT and liver were removed and weighed. Portions were snap-frozen in liquid nitrogen for RNA or protein extraction or processed for histology.

### Indirect calorimetry

After 10 weeks of HFD feeding, male WT and KO ADAMTS5-J mice were individually housed in automated Calocages for indirect calorimetry (PhenoMaster CaloCages; TSE systems, Bad Homburg, Germany) during 72 h on a 12h-dark/light cycle at 22°C with *ad libitum* access to HFD and water. Prior to actual measurements, mice were adapted in regular filter-top cages for 7 days to single housing and specific drinking nipples, followed by a 48 h adaptation period in the Calocages. Food intake, oxygen consumption (VO_2_), carbon dioxide production (VCO_2_), heat generation and ambulatory activity were continuously recorded over a 24 h period. Respiratory exchange ratio (RER = VCO_2_ / VO_2_) and energy expenditure (EE = 1.44 x RER (3.815 + 1.232 x VO_2_)) were calculated [17]. Spontaneous locomotive activity was defined as total counts of infrared light beam breaks along the X-Y axes.

### Gene expression analysis

DNA-free total RNA was extracted using the RNAeasy kit (Qiagen, Basel, Switzerland) according to the manufacturer’s instructions. RNA concentrations were measured spectrophotometrically and total RNA samples were stored at -80°C. Complementary DNA was prepared from total RNA using the TaqMan^®^ Reverse Transcription Reagents (Applied Biosystems, Foster City, CA, USA). PCR reactions were performed from 10 ng/μl total RNA at 25°C for 10 min, followed by amplification at 48°C for 1 h and finally 5 min at 95°C. Quantitative real time PCR was performed in the ABI 7500 Fast Sequence detector using the TaqMan^®^ Fast Universal PCR Master Mix and TaqMan^®^ Gene Expression Assays (Applied Biosystems), reported elsewhere [12, 14]. Fold differences in gene expression were calculated with the ΔΔCt method, using β-actin as housekeeping gene (http://dx.doi.org/10.17504/protocols.io.kbgcsjw).

### Western blotting

AT samples were homogenized with a FastPrep Ribolyser (MP Biomedicals, Elsene, Belgium) in 10 mM Na phosphate, pH 7.2, 150 mM NaCl, 1% Triton, 0.1% sodium dodecyl sulphate (SDS), 0.5% Na deoxycholate, 0.2% NaN_3_, containing a protease inhibitor cocktail (Thermo Fisher Scientific, Rockford, IL, USA). Protein concentrations were determined with the BCA protein assay (Thermo Fisher Scientific, Rockford, IL, USA). An equal amount of protein was loaded in each well of a 10% SDS-PAGE (Bio-Rad, Hercules, CA, USA). Gels were transferred onto a 0.45 μm nitrocellulose membrane and blocked in 5% nonfat dry milk (Bio-Rad) in 10 mM Tris–HCl buffer containing 150 mM NaCl and 0.05% Tween 20 at pH 7.6 (TBST) for 3 h. Subsequently, membranes were probed with the following primary antibodies: β-actin (Cell Signaling Technology; CST, Leiden, The Netherlands), glyceraldehyde 3-phosphate dehydrogenase (GAPDH, CST), peroxisome proliferator-activated receptor gamma coactivator 1 α (PGC1α, Novus Biologicals, Abingdon, UK), and UCP-1 (Sigma-Aldrich, Darmstadt, Germany). Secondary antibodies were species-appropriate horseradish peroxidase-conjugated antibodies (Dako, Glostrup, Denmark, 1:2000) diluted in TBST containing 5% nonfat dry milk. Signals were detected with Enhanced Chemiluminescence (Thermo Fisher Scientific) (http://dx.doi.org/10.17504/protocols.io.kbfcsjn).

### Biochemical analysis

Metabolic parameters were determined using standard clinical laboratory assays (http://dx.doi.org/10.17504/protocols.io.j9dcr26). Fasting insulin was measured using a commercial ELISA (Mercodia, Uppsala, Sweden) and glucose with the Accu-chek performa meter and blood glucose test strips. The homeostasis model assessment of insulin resistance (HOMA-IR) was calculated using the formula: [plasma insulin (ng/ml) x blood glucose (mg/dl)]/405. Hepatic triglyceride content was quantified with the FS* kit (Diasys, Holzheim, Germany), as described [14]. sWAT sections (http://dx.doi.org/10.17504/protocols.io.katcsen) were stained with haematoxylin-eosin (H&E) to detect the presence of brown adipocytes in these sections (http://dx.doi.org/10.17504/protocols.io.j9qcr5w). This was confirmed by staining AT sections for UCP-1 using a polyclonal rabbit anti-mouse UCP-1 antibody (1:200, ab10983, Abcam, Cambridge, UK), followed by biotin-coupled goat anti-rabbit secondary antibody (1:300, Dako Cytomation, Heverlee, Belgium) and a TSA biotinyl tyramide amplication kit (NEL700001, Perkin Elmer, Boston, MA) (http://dx.doi.org/10.17504/protocols.io.j9scr6e). Images were taken of the H&E and UCP-1 stained sWAT sections using a Axiovert 200M microscope (Zeiss, Jena, Germany) and the Axiovision Rel. 4.8.2. software (Zeiss) at a magnification of 400x. Liver sections (http://dx.doi.org/10.17504/protocols.io.katcsen) were stained with H&E or Sirius Red (http://dx.doi.org/10.17504/protocols.io.j9rcr56) to assess steatosis and fibrosis, respectively [14]. In addition, foci of inflammation and ballooning (swelling and enlargement of hepatocytes) were determined on H&E stained sections. The NAFLD Activity Score (NAS) was determined as the addition of semi quantitative scores of steatosis (from 0 to 3), ballooning (from 0 to 2) and inflammation (from 0 to 3), according to Kleiner *et al* [18].

### Statistical analysis

Data are expressed as means ± SEM. Differences between two groups were analyzed with the non-parametric Mann-Whitney U test, compatible with small sample sizes. Analysis was done with Prism 5 (GraphPad Software Inc., San Diego, CA, USA). Two-sided values of p < 0.05 are considered statistically significant.

## Results

### Characterization of ADAMTS5-P and ADAMTS5-J mice

Heterozygous breeding pairs of ADAMTS5-J mice (26 couples monitored) produced on average 5.5 ± 0.5 pups per litter with a mean time of 33 ± 1.7 days between litters; corresponding values for ADAMTS5-P mice (14 couples) were 6.4 ± 0.6 pups and 31 ± 2.0 days. Gender distribution was equal, with 51% male and 49% female for ADAMTS5-P *versus* 52% and 48% for ADAMTST5-J mice. For 295 ADAMTS5-P and 306 ADAMTS5-J mice that were genotyped, the genotype distribution followed the expected inheritance pattern for both males and females (Table 1). The somewhat skewed distribution for the ADAMTS5-J mice may be due to a higher mortality of the KO offspring.

**Table 1:** Genotype distribution of littermates obtained by interbreeding heterozygous deficient ADAMTS5 mice.

	Wild-type	Heterozygous	Homozygous	Total
	n (%)	n (%)	n (%)	n
**ADAMTS5-J**				
Male	61 (32)	95 (50)	35 (18)	191
Female	37 (31)	60 (52)	18 (17)	115
Total	98 (31)	155 (51)	53 (18)	306
**ADAMTS5-P**				
Male	43 (23)	95 (52)	45 (25)	183
Female	26 (23)	52 (46)	34 (30)	112
Total	69 (23)	147 (49)	79 (27)	295

**Fig 1:**
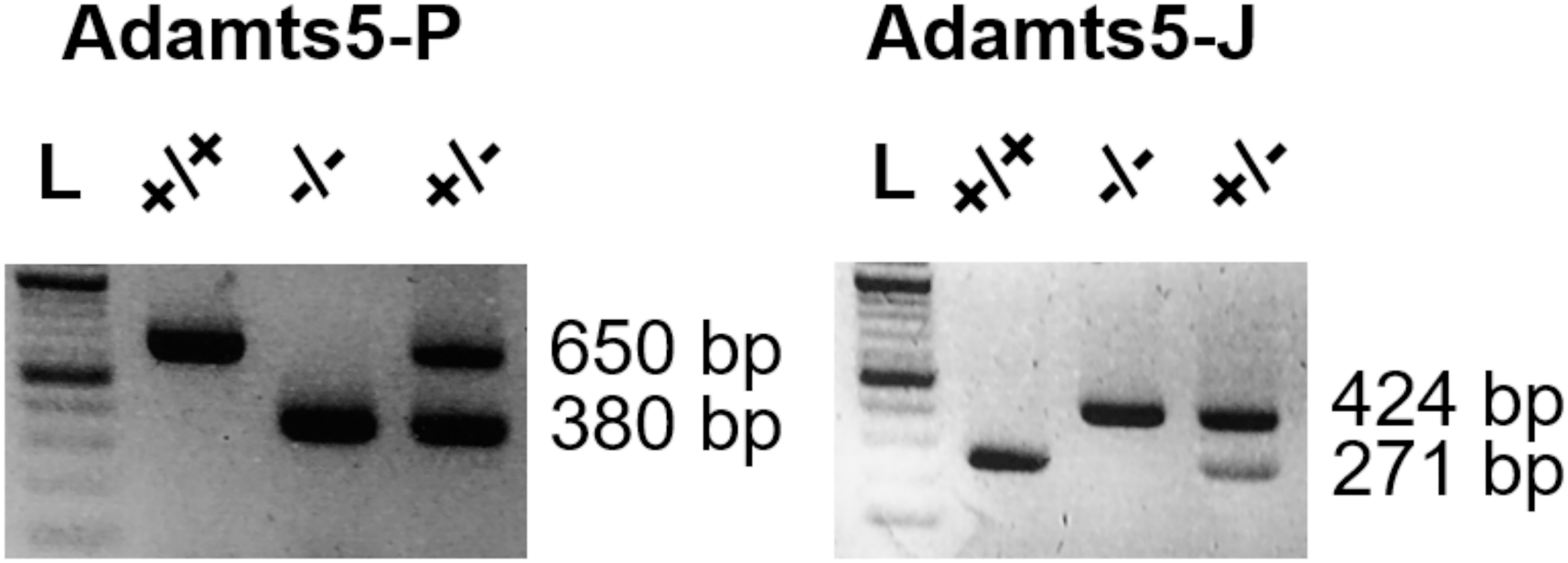
Characterization of ADAMTS5-P and ADAMTS5-J mice. Genotyping of wild-type (+/+), heterozygous (+/-) and homozygous (-/-) deficient ADAMTS5-P and ADAMTS5-J mice. L represents the basepair (bp) ladder. ADAMTS5, a disintegrin and metalloproteinase with thrombospondin type 1 motifs member 5.

At 8 weeks of age, body weights of male ADAMTS5-J WT and KO mice (Table 2) were indistinguishable from their ADAMTS5-P counterparts [11]. Both strains were genotyped as described [13, 15] yielding the expected fragments at 650 bp or 380 bp for WT or KO ADAMTS5-P mice, with corresponding bands at 271 bp or 424 bp for WT or KO ADAMTS5-J mice (Fig 1).

**Table 2:** Effect of ADAMTS5 deficiency on body and organ weights and fat mass of mice kept on a high-fat diet (HFD) for 15 weeks.

	Wild-type	*Adamts5*^-/-^-J
	(n = 6)	(n = 7)
BW start (g)	26.5 ± 1.0	24.9 ± 0.4
BW end (g)	40.4 ± 1.8	35.1 ± 1.3
Food intake (g/day)	3.8 ± 0.1	3.7 ± 0.1
sWAT (mg/g)	26.4 ± 2.5	20.4 ± 2.7
GON fat (mg/g)	48.9 ± 3.0	36.4 ± 4.5^a^
BAT (mg/g)	4.3 ± 0.3	3.7 ± 0.3
Liver (mg/g)	57.6 ± 8.4	36.3 ± 2.4^a^
Heart (mg/g)	4.2 ± 0.1	4.8 ± 0.4

Data are means ± SEM. BW, body weight; s, subcutaneous; GON, gonadal; BAT, brown adipose tissue; WAT, white adipose tissue.

^a^p<0.05 versus wild-type mice according to the non-parametric Mann-Whitney U test.

### Effect of ADAMTS5 deficiency on adipose tissue

**Fig 2:**
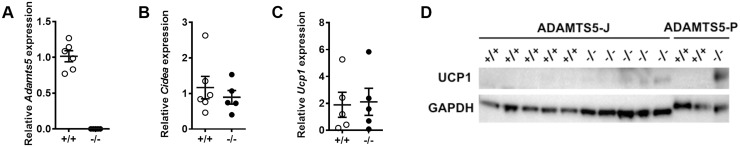
Effect of ADAMTS5 deficiency on diet-induced obesity. (A-C) Expression of *Adamts5* (A disintegrin and metalloproteinase with thrombospondin type 1 motifs member 5), *Ucp-1* (Uncoupling protein-1) and *Cidea* in subcutaneous (s) white adipose tissue (WAT) of ADAMTS5-J mice (n = 5–6). Data are corrected for the housekeeping gene *β-actin* and normalized to wild-type (WT) mice (+/+). Data are means ± SEM of n determinations. (D) Western blot analysis of UCP-1 protein levels in sWAT of *Adamts5*^+/+^-P (n = 2), *Adamts5*^-/-^-P (-/-; n = 1), *Adamts5*^+/+^-J (n = 5) and *Adamts5*^-/-^-J (-/-; n = 5) mice. The expression of the housekeeping gene glyceraldehyde 3-phosphate dehydrogenase (GAPDH) was used as a loading control.

ADAMTS5-J homozygous deficient and WT mice showed comparable body weights before and after (p = 0.08) 15 weeks of HFD feeding. Food intake was comparable for both genotypes (Table 2). After 15 weeks, no significant differences were observed in sWAT or GON WAT, nor BAT mass (Table 2). Gene expression analysis in sWAT confirmed the ADAMTS5 KO genotype (Fig 2A). Expression of *Cidea* or *Ucp1* was, however, not different between WT and *Adamts5*^-/-^-J strains (Fig 2B and 2C). UCP-1 protein was around the detection limit of Western blotting for WT ADAMTS5-J samples (as also observed for WT ADAMTS5-P mice), but also for *Adamts5*^-/-^-J samples (in contrast to *Adamts5*^-/-^-P mice) (Fig 2D).

Analysis of plasma metabolic parameters did not reveal significant differences in glucose, cholesterol or triglyceride levels between both strains. Insulin levels were lower, resulting in a reduction of the HOMA-IR by 50% (Table 3).

**Table 3:** Effect of ADAMTS5 deficiency on metabolic parameters of mice kept on a high-fat diet (HFD) for 15 weeks.

	Wild-type	*Adamts5*^-/-^-J
Blood glucose (mg/dl)	164 ± 9.6	136 ± 16
Insulin (ng/ml)	2.3 ± 0.7	1.3 ± 0.4
HOMA-IR (ng/ml x mg/dl)	1.0 ± 0.3	0.5 ± 0.1
Total cholesterol (mg/dl)	158 ± 12	160 ± 7
HDL-cholesterol (mg/dl)	139 ± 10	149 ± 6
Non-HDL-cholesterol (mg/dl)	18 ± 2	11 ± 2
AP (U/I)	72 ± 10	51 ± 3
AST (U/l)	92 ± 27	82 ± 13
ALT (U/I)	87 ± 43	69 ± 14
Triglycerides (mg/dl)	41 ± 4	42 ± 3

Data are means ± SEM of 6–7 determinations for glucose and insulin levels. Data are means ± SEM of 4–5 determinations for cholesterol, triglycerides and liver enzymes. AP, alkaline phosphatases; AST, aspartate aminotransferase; ALT, alanine aminotransferase; HOMA-IR, homeostatic model assessment of insulin resistance and HDL, high density lipoproteins.

**Fig 3:**
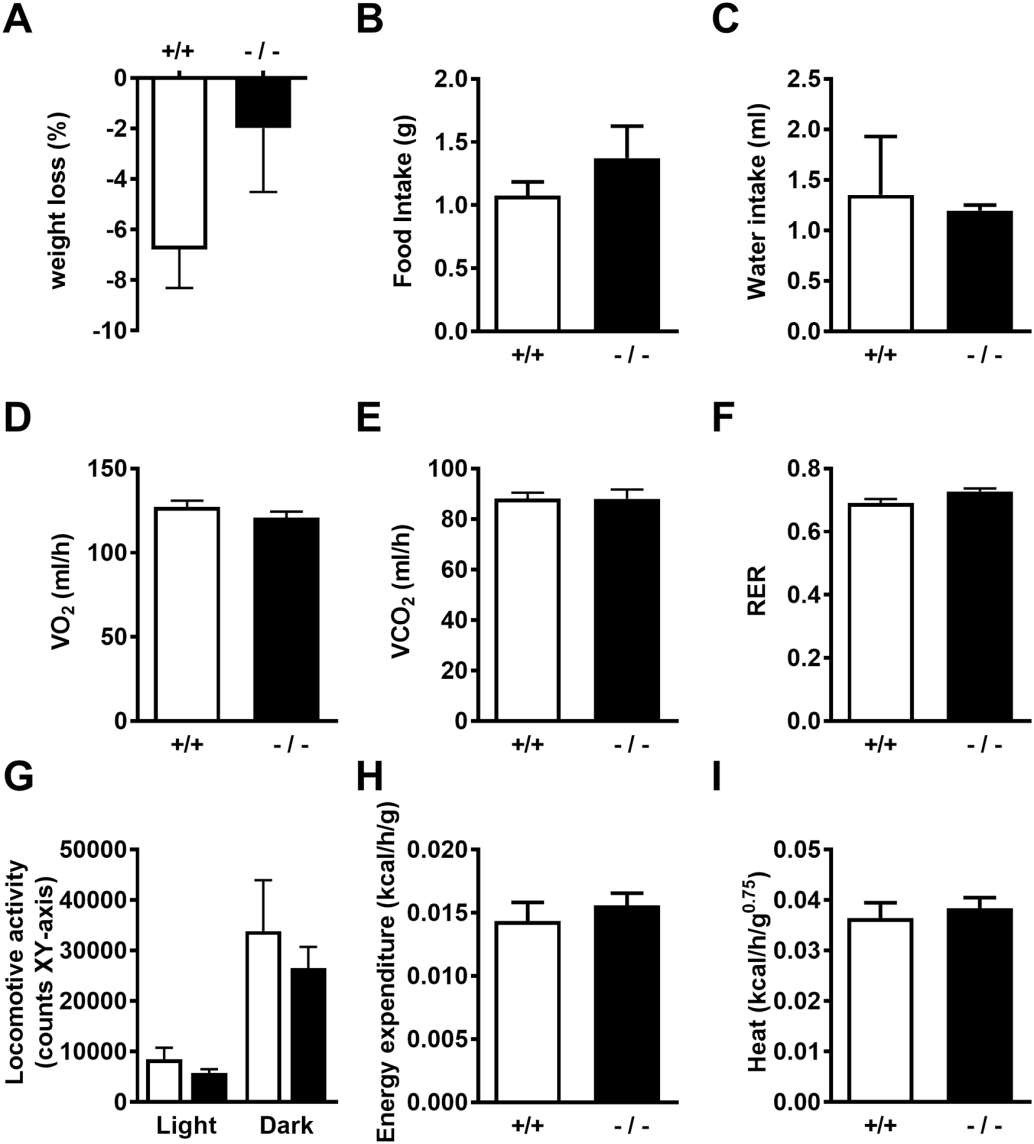
Indirect calorimetry with ADAMTS5-J mice after 10 weeks of HFD feeding. (A) Body weight change of wild-type (white bars; +/+) and *Adamts5*^-/-^-J mice (black bars; -/-) over 72 h. (B,C) Food and water intake. (D,E) Oxygen consumption (VO2) and carbon dioxide production (VCO2). (F) Calculated respiratory exchange rate (RER). (G) Locomotive activity during dark and light phase. (H,I) Energy expenditure and heat production over 24 h. Data are means ± SEM of 3 determinations for all panels. ADAMTS5, a disintegrin and metalloproteinase with thrombospondin type 1 motifs member 5; HFD, high-fat diet.

Indirect calorimetry with ADAMTS5-J mice after 10 weeks of HFD feeding revealed weight loss for WT as well as KO mice during the 72 h experimental period (Fig 3A). Food (Fig 3B) and water (Fig 3C) intake, as well as VO_2_, VCO_2_ and calculated RER were not different between genotypes (Fig 3D–3F). Locomotive activity was higher during phases of darkness, but was not different between genotypes (Fig 3G). Energy expenditure (Fig 3H) and heat production (Fig 3I) were also not significantly different.

### Effect of cold exposure on browning of WAT

**Fig 4:**
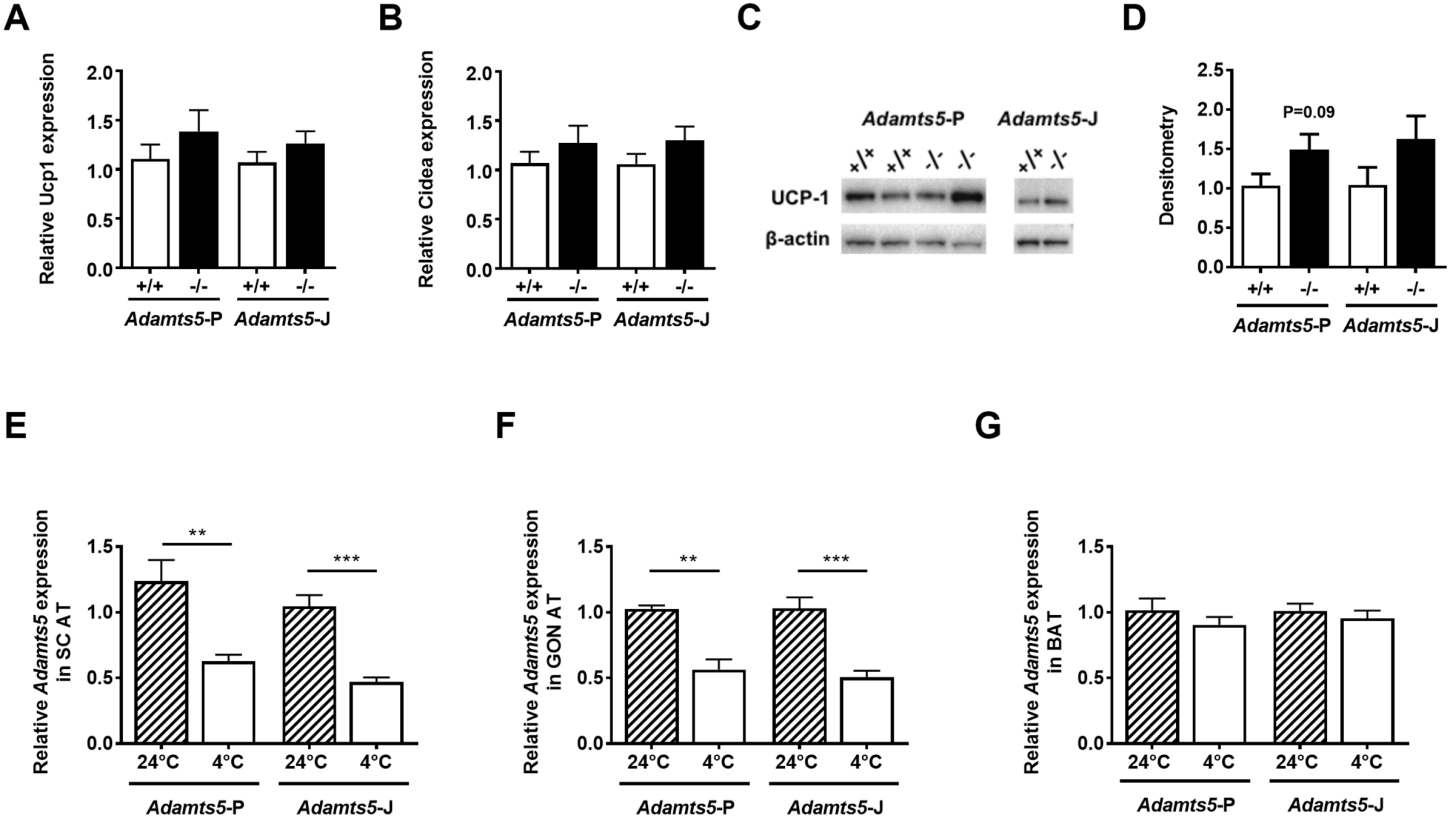
Effect of ADAMTS5 genotype on AT during cold exposure for 72 h. (A, B) Gene expression levels of *Ucp-1* (A) and *Cidea* (B) in sWAT of WT (+/+) and homozygous deficient (-/-) mice from the ADAMTS5-P (+/+: n = 10; -/-: n = 8) and ADAMTS5-J strains (+/+: n = 11; -/-: n = 7). Gene expression levels are normalized to the housekeeping gene *Tbp* and shown relative to WT mice at +4°C. (C,D) UCP-1 protein levels in sWAT. The Western blots (C) are quantitated by densitometry, normalized to β-actin and expressed relative to WT (D). (E-G) Relative ADAMTS5 gene expression in sWAT (E), GON AT (F) and BAT (G) of WT mice kept at 24°C or at 4°C. Gene expression levels are normalized to the housekeeping gene *Tbp* and shown relative to WT mice at 24°C. Data are means ± SEM of 4–8 and 5–11 determinations for panel D and panel E-G, respectively. ** p<0.01, *** p<0.001 versus 24°C according to the non-parametric Mann-Whitney U test. ADAMTS5, a disintegrin and metalloproteinase with thrombospondin type 1 motifs member 5; WAT, white adipose tissue; UCP-1, uncoupling protein-1; s, subcutaneous; WT, wild-type; GON, gonadal; BAT, brown adipose tissue and *Tbp*, TATA-box binding protein.

During exposure of WT or homozygous deficient ADAMTS5-P and ADAMTS5-J mice to 4°C for only 72 h, all mice lost some weight, whereas food intake was not different between genotypes (Table 4). There was no significant effect of ADAMTS5 deficiency on sWAT or GON WAT mass of either strain. Whereas for the *Adamts5*^-/-^-P mice the BAT mass was significantly higher than for WT controls, this was not observed for the *Adamts5*^-/-^-J mice. The expression levels of *Ucp1* or *Cidea* in sWAT of ADAMTS5 deficient mice of either strain were also not significantly different from WT controls (Fig 4A and 4B). Representation of *Ucp-1* mRNA levels relative to the *Adamts5*^+/+^-P *Ucp-1* expression levels did not induce statistically significant differences between the four groups (*Adamts5*^*+/+*^-P: 1.11 ± 0.15, *Adamts5*^*-/—*^P: 1.39 ± 0.22, *Adamts5*^*+/+*^-J: 0.82 ± 0.09 and *Adamts5*^*-/-*^-J: 0.97 ± 0.09; p>0.05). Western blotting for UCP-1 revealed slightly elevated protein levels in sWAT of both KO strains (Fig 4C and 4D). *Adamts5*^-/-^-J versus *Adamts5*^-/-^-P sWAT protein extracts showed reduced UCP-1 protein levels (p = 0.02) when levels were expressed relative to *Adamts5*^+/+^-P levels (*Adamts5*^+/+^-P: 1.04 ± 0.15, *Adamts5*^-/-^-P: 1.49 ± 0.20, *Adamts5*^+/+^-J: 0.62 ± 0.13 and *Adamts5*^-/-^-J: 0.67 ± 0.14). Interestingly, *Adamts5* gene expression was significantly lower in sWAT and GON AT of WT mice kept at 4°C as compared to 24°C (Fig 4E and 4F), whereas this was not observed for BAT (Fig 4G).

**Table 4:** Effect of ADAMTS5 genotype on body and tissue weight in mice exposed to cold for 72 h.

	ADAMTS5-P		ADAMTS5-J	
	+/+	-/-	+/+	-/-
	n = 10	n = 8	n = 11	n = 7
BW start (g)	26.7 ± 0.5	26.8 ± 0.7	25.2 ± 0.2	25.2 ± 0.6
BW end (g)	25.7 ± 0.6	26.1 ± 0.8	23.6 ± 0.3	24.1 ± 0.6
BW change (%)	-3.7 ± 0.9	-2.8 ± 0.5	-6.1 ± 0.8	-4.3 ± 0.5
FI (g/day)	8.0 ± 0.4	8.0 ± 0.2	6.7 ± 0.3	6.7 ± 0.3
sWAT (mg)	175 ± 10.0	187 ± 17.4	142 ± 6.3	140 ± 10.1
GON fat (mg)	250 ± 17.8	251 ± 19.4	201 ± 7.6	194 ± 13.5
BAT (mg)	79 ± 2.0	92 ± 3.0^a^	63 ± 1.3	63 ± 2.4

Data are means ± SEM. BW, body weight; FI, food intake; s, subcutaneous; GON, gonadal; BAT, brown adipose tissue; WAT, white adipose tissue.

^a^ p<0.01 *versus* the corresponding wild-type (+/+) controls according to the non-parametric Mann-Whitney U test.

**Fig 5:**
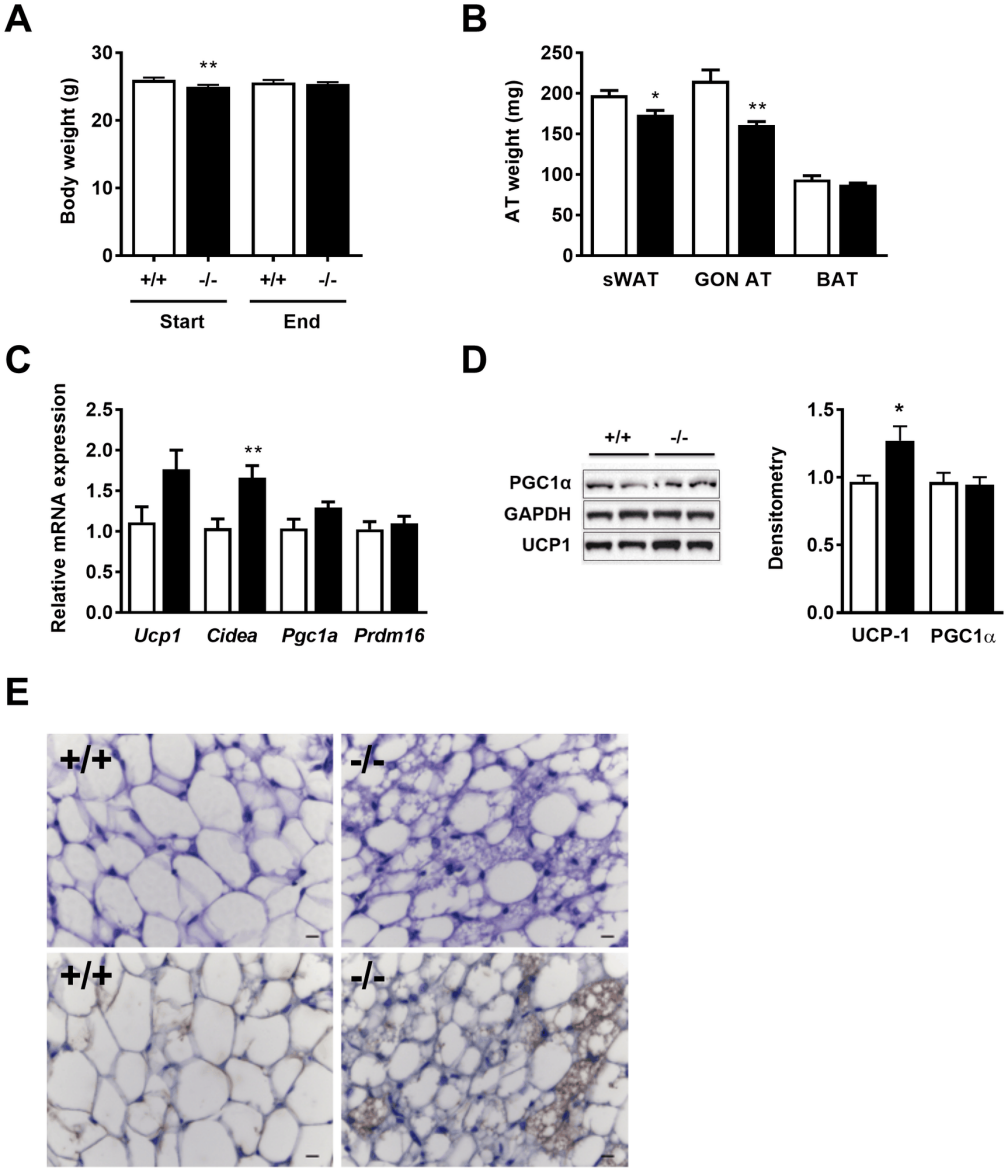
Effect of ADAMTS5 genotype on AT after cold exposure for 2 weeks. (A) Body weight of ADAMTS5-J WT (+/+; n = 7) or homozygous deficient (-/-; n = 9) mice at start and end of the experiment. (B) Mass of sWAT, GON AT and BAT for WT (white bars; n = 8) and *Adamts5*^-/-^-J mice (black bars; n = 9). (C) Gene expression levels of markers of browning of sWAT, normalized to the housekeeping gene *Tbp* and shown relative to WT (n = 7–8). (D) sWAT western blots for UCP-1 (n = 7–8) and PGC1α (n = 7–9), and quantitation by densitometry, normalized to GAPDH and shown relative to WT. (E) sWAT sections of WT (+/+) and *Adamts5*^-/-^ -J mice were stained with haematoxylin & eosin (upper panel) or with antibodies directed against UCP-1 (lower panel; brown color) to illustrate browning of sWAT. Magnification: 400x. Scale bar = 10 μm. Data are means ± SEM of n determinations. * p<0.05, ** p<0.01 *versus* WT mice according to the non-parametric Mann-Whitney U test. ADAMTS5, a disintegrin and metalloproteinase with thrombospondin type 1 motifs member 5; WAT, white adipose tissue; UCP-1, uncoupling protein-1; s, subcutaneous; WT, wild-type; GON, gonadal; BAT, brown adipose tissue; *Tbp*, TATA-box binding protein; PGC1α, peroxisome proliferator-activated receptor gamma coactivator 1-α and GAPDH, glyceraldehyde 3-phosphate.

In our previous study with ADAMTS5-P mice exposed to cold for 2 weeks, we observed for the KO as compared to the WT mice, (i) a significantly higher BAT mass, (ii) loss of body weight, and (iii) markedly higher expression of UCP-1 in sWAT. Therefore, we have also exposed WT and *Adamts5*^-/-^-J mice to 4°C for 2 weeks. Whereas sWAT and GON AT mass at the end of the experiments were significantly lower for KO mice, no effect was observed on total body weight or BAT mass (Fig 5A and 5B). Expression of the browning markers *Ucp1*, *Cidea* and *Pgc1α*, but not *PR domain-containing 16* (*Prdm16)*, was slightly enhanced for KO mice (Fig 5C). Western blot analysis confirmed enhanced UCP-1 protein levels in sWAT of *Adamts5*^-/-^-J mice, as compared to WT mice (Fig 5D). Fig 5E illustrates the enhanced sWAT browning of *Adamts5*^-/-^-J versus WT mice in sections stained with H&E or UCP-1 antibodies.

### Effect of ADAMTS5 deficiency on liver function

**Fig 6:**
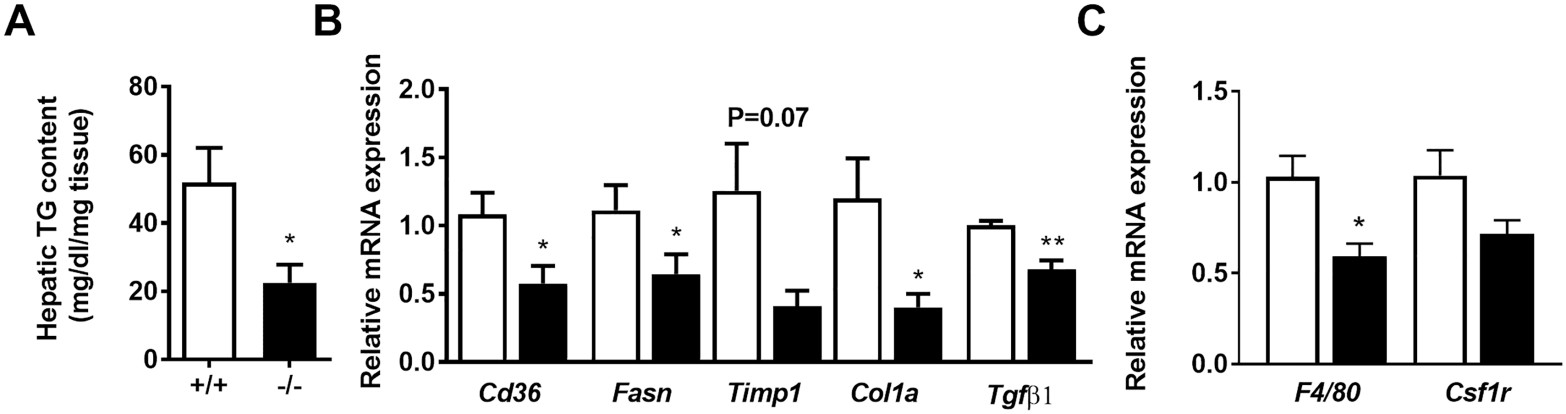
Liver characterization of WT (+/+) and *Adamts5*^-/-^-J (-/-) mice kept on HFD for 15 weeks. (A) Hepatic triglyceride (TG) content. (B) Gene expression levels of markers for steatosis and fibrosis and (C) of pan-macrophage markers in livers of obese WT (white bars) and *Adamts5*^-/-^-J (black bars) mice were normalized to the housekeeping gene *β-actin* and shown relative to WT. Data are means ± SEM of 6 (WT) or 7 (*Adamts5*^-/-^-J) experiments for all panels. *p≤0.05 and **p<0.005 *versus* WT mice by non-parametric Mann-Whitney U test. ADAMTS5, a disintegrin and metalloproteinase with thrombospondin type 1 motifs member 5; WT, wild-type; HFD, high-fat diet; *Cd36*, Cluster of differentiation 36; *Fasn*, Fatty acid synthase; *Timp1*,Ttissue inhibitor of metalloproteinases 1; *Col1a*, Collagen type 1; *Tgfβ*_*1*_, Transforming growth factor β_1_ and *Csf1r*, Colony stimulating factor 1 receptor.

After 15 weeks of HFD feeding, liver weight (Table 2) and hepatic triglyceride content (Fig 6A) were significantly lower for KO mice. Plasma triglyceride levels were, however, comparable for WT and KO mice (Table 3). Liver enzymes including alkaline phosphatases, aspartate aminotransferase and alanine aminotransferase were non-significantly lower for the *Adamts5*^-/-^-J mice (Table 3).

**Fig 7:**
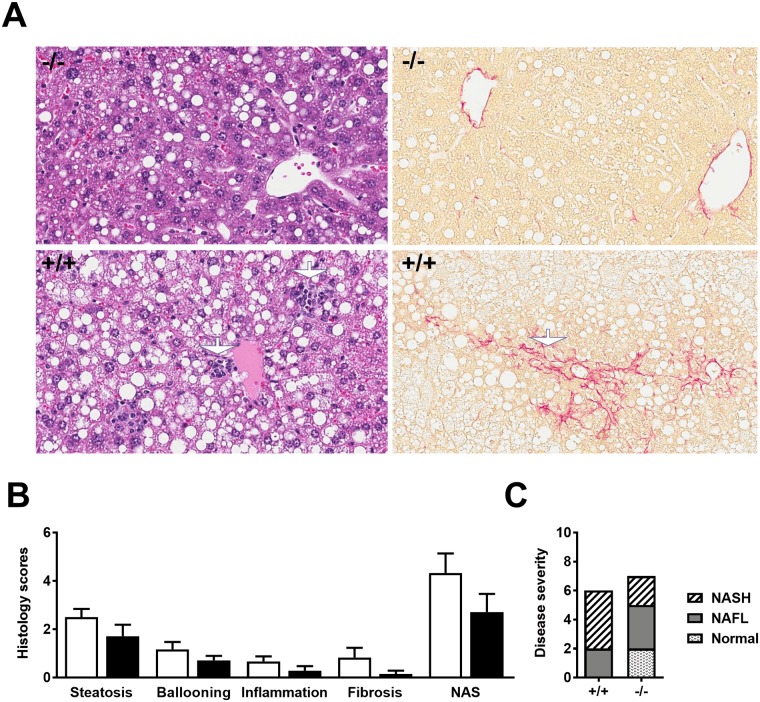
Histological analysis of liver sections of WT (+/+) and *Adamts5*^-/-^-J (-/-) mice kept on HFD for 15 weeks. (A) Representative Haematoxylin & Eosin (H&E) (left) and Sirius Red (right) stainings. Magnification: 200x. The white arrows indicate foci of inflammation in the H&E stainings and sinusoidal fibrosis in the Sirius Red stainings. (B) Histological scoring of steatosis, hepatocyte ballooning, inflammation, fibrosis and non-alcoholic fatty liver (NALF) activity score (NAS). (C) Disease severity distribution, as number of mice without or with NALF/NASH. Data are means ± SEM of 6 (WT) or 7 (*Adamts5*^-/-^-J) experiments for panels B and C. ADAMTS5, a disintegrin and metalloproteinase with thrombospondin type 1 motifs member 5; WT, wild-type; HFD, high-fat diet and NASH, non-alcoholic steatohepatitis.

Liver expression of the steatosis markers *Cd36* (Cluster of differentiation 36) and *Fasn (*Fatty acid synthase) and the fibrosis markers *Timp1* (Tissue inhibitor of metalloproteinases 1), *Col1a1* (Collagen type 1) and *Tgfβ1* (Transforming growth factor β_1_) was significantly lower for *Adamts5*^-/-^-J as compared to WT mice (Fig 6B). In addition, ADAMTS5 deficient animals showed reduced mRNA expression levels of the pan-macrophage markers *F4/80* and *Csf1r* (Colony stimulating factor 1 receptor) in liver tissues as compared to WT control littermates (Fig 6C). However, analysis of M1 (*Tnf-α* (Tumour necrosis factor-α); *Il-1β* (Interleukin-1β) and *Mcp-1* (Monocyte chemoattractant protein-1)) and M2 macrophage markers (*Arg1* (Arginase 1) and *Mrc1* (Mannose receptor c type 1)) did not show significant differences in gene expression between both groups (S1 Fig). Quantitative analysis of H&E and Sirius Red stainings of liver sections (Fig 7A) confirmed reduced steatosis, hepatocyte ballooning, inflammation and fibrosis, resulting in a lower NAS score for KO mice (Fig 7B). This is in accordance with the disease severity distribution (Fig 7C).

These differences between WT and *Adamts5*^-/-^-J mice are comparable, although somewhat less pronounced, to those observed in our previous study in *Adamts5*^-/-^-P mice [14], thus confirming specificity of the phenotype.

## Discussion

Previous studies comparing *Adamts5*^-/-^-P [13] and WT mice kept on HFD revealed: (i) reduced WAT mass, (ii) increased BAT mass, (iii) enhanced browning of WAT, (iv) enhanced insulin sensitivity, and (v) protection against NASH for KO mice [11, 12, 14]. Because frequently divergent results are reported in studies with gene-deficient mice, we have substantiated these observations using an independently generated strain of ADAMTS5 deficient mice (*Adamts5*^-/-^-J) [15].

Comparison of the phenotype of the *Adamts5*^-/-^-J mice of the present study with that of the *Adamts5*^-/-^-P mice reveals some similarities supporting specificity of the phenotype, but also some quantitative and qualitative differences: (i) with respect to WAT mass: in *Adamts5*^-/-^-J mice lower sWAT and GON fat mass was found, which was significant after 2 weeks of cold exposure, but only showed a trend in the DIO model; (ii) with respect to BAT mass: in *Adamts5*^-/-^-J mice no significant difference with WT mice was observed after 2 weeks of cold exposure nor in the DIO model; (iii) with respect to browning of WAT: in *Adamts5*^-/-^-J mice higher expression of UCP-1 mRNA and protein was found after 2 weeks of cold exposure (mRNA: 1.5- to 2-fold as compared to 5-fold for *Adamts5*^-/-^-P mice). Furthermore, cold exposure for 72h led to lower UCP-1 protein levels (relative to ADAMTS5-P WT) in *Adamts5*^-/-^-J versus *Adamts5*^-/-^-P sWAT extracts. However, in the DIO model UCP-1 or Cidea expression levels in sWAT were not different between WT and *Adamts5*^-/-^ -J mice (as compared to 30-fold enhancement for *Ucp1* or 5-fold for *Cidea* for *Adamts5*^-/-^-P versus WT) [12]. Indirect calorimetry did not reveal differences in VO_2_, VCO_2_ or RER (same as for *Adamts5*^-/-^-P mice), and no effect of genotype was observed on energy expenditure or heat production, in contrast to *Adamts5*^-/-^-P mice [12]. These observations are in agreement with the finding that BAT mass is only decreased in ADAMTS5^-/-^-P and not in ADAMTS5^-/-^-J mice as compared to their respective control littermates. In addition, (iv) with respect to insulin sensitivity: for *Adamts5*^-/-^-J mice a 50% lower HOMA-IR was found in the DIO model as compared to WT mice, as also seen in the *Adamts5*^-/-^-P mice (which was confirmed by insulin tolerance tests) [14], and (v) with respect to NASH: *Adamts5*^-/-^-J mice had lower liver weight and hepatic triglyceride content and developed less steatosis or fibrosis, as shown by histological stainings and expression of marker genes (as observed for the *Adamts5*^-/-^-P mice). Furthermore, with respect to the hepatic inflammatory profile: both KO strains showed reduced mRNA levels of the pan-macrophage markers *F4/80* and *Csf1r*.

Thus, ADAMTS5 deficiency is associated with reduced WAT mass in both KO strains, and with enhanced BAT mass only in *Adamts5*^-/-^-P mice. Browning of WAT is observed in both strains upon cold exposure, but only for the ADAMTS5^-/-^-P strain in the DIO model (less stringent condition). The activated thermogenic profile in *Adamts5*^-/-^-P mice was due to an increased activation of the β_3_-AR signaling pathway via protein kinase A and CREB [12]. Therefore, one possible mechanism for the lack of diet-induced sWAT browning in *ADAMTS5*^*-/-*^-J mice may be a lower activation of the β_3_-AR pathway. Insulin sensitivity is improved in both KO strains, and both strains are protected against NASH.

Specificity of the ADAMTS5-P phenotype is further supported by our finding that *in vitro* differentiation into mature adipocytes of 3T3-F442A preadipocytes with stable *Adamts5* gene silencing, or of embryonic fibroblasts derived from *Adamts5*^-/-^-P mice, was significantly impaired as compared to control cells [11]. *De novo* fat pad formation following injection of 3T3-F442A cells with *Adamts5* knockdown in NUDE mice was also significantly reduced as compared to controls [11]. Furthermore, the DIO studies in *Adamts5*^-/-^-P mice were performed in total in 60 WT and 54 KO mice on HFD, in several independent studies conducted over a 3 year period. In addition, similar data were obtained using a different diet composition [14].

There may be several explanations for the quantitative differences observed between both ADAMTS5 KO strains. For both sets of studies true littermates were used, all males of comparable age and body weight and all correctly genotyped. Housing conditions and diets were also comparable. Although we have thus done every effort to perform the studies with both strains under comparable external and experimental conditions, we can not exclude differences in microbiota. It is indeed known that the bacterial composition of the gut can significantly affect the metabolic state [19, 20]. The small difference in genetic background of both strains (99.6% C57Bl/6J plus 129S5 plus FVB/N for *Adamts5*^-/-^-P *versus* 100% C57Bl/6J for *Adamts5*^-/-^-J [13, 15, 21]) may also contribute, as it is known that this strongly affects DIO and metabolism in mice [22, 23]. Also, modifier genes (i.e. inherited genetic variations leading to qualitative and quantitative differences in disease phenotype) may be variably expressed in different inbred mouse strains [24, 25]. Furthermore, differences between KO and WT mice may become more apparent using stronger stimuli, such as prolonged cold exposure, different diet compositions, prolonged monitoring in Calocages,… It is also possible that during the KO procedure another gene locus has been affected, which may result in activation/inactivation of a confounding gene. To generate the *Adamts5*^-/-^-P mice, exon 2 (encoding part of the proteolytic domain) was flanked by loxP sites, in combination with Cre-recombinase under control of the Ella promoter [13]. For the *Adamts5*^-/-^-J mice, the targeting construct containing an IRES-lacZ-neomycin cassette, substituted 134 nucleotides of exon 2, including the catalytic site [15]. Although the KO in both strains thus results in inactivation of the enzymatic activity, it remains unclear whether a truncated form of the protein may be expressed. This may affect signaling pathways (e.g. CREB mediated [12]) that are involved in UCP-1 expression. Alternatively, Gorski *et al*. reported the presence of a truncated ADAMTS5 species (34–40 kDa) in muscle extracts of WT mice that was undetectable in equivalent KO samples (both *Adamts5*^-/-^-P and *Adamts5*^-/-^-J) [16]. Some functions of ADAMTS5 may indeed be independent of its proteolytic activity. Thus, an anti-angiogenic and anti-tumorigenic action has been reported mediated via the first thrombospondin type 1 repeat, independent of the proteoglycanase activity [26, 27]. A non-proteolytic role of ADAMTS5 has also been proposed to explain its anti-chondrogenic and pro-fibrotic effects in murine models of wound repair [16].

In any case, our observations strongly suggest that data obtained in a single strain of KO mice should be interpreted with great care. Ideally, a phenotype rescue by (tissue)specific (over)expression of the missing component should confirm specificity. In addition, a tissue-specific KO or *in vivo* gene silencing of the target may help to confirm the phenotype.

## Supporting information

S1 Fig Macrophage identification in livers of WT (+/+) and *Adamts5*^-/-^-J (-/-) mice kept on HFD for 15 weeks. Gene expression levels of M1 macrophage markers (*Tnf-α* (Tumour necrosis factor-α); *Il-1β* (Interleukin-1β) and *Mcp-1* (Monocyte chemoattractant protein-1)) and M2 macrophage markers (*Arg1* (Arginase 1) and *Mrc1* (Mannose receptor c type 1)) in livers of obese WT (white bars) and *Adamts5*^-/-^-J (black bars) mice were normalized to the housekeeping gene *β-actin* and shown relative to WT. Data are means ± SEM of 6 (WT) or 7 (*Adamts5*^-/-^-J) experiments. ADAMTS5, a disintegrin and metalloproteinase with thrombospondin type 1 motifs member 5; WT, wild-type; HFD, high-fat diet.
(TIF)

S1 Data Compressed zip file of all raw data. (ZIP)

## References

[1] Townsend K, Tseng YH. Brown adipose tissue: Recent insights into development, metabolic function and therapeutic potential. Adipocyte. 2012;1: 13–24. doi: 10.4161/adip.18951 23700507 10.4161/adip.18951 PMC3661118

[2] Lidell ME, Betz MJ, Enerback S. Brown adipose tissue and its therapeutic potential. J Intern Med. 2014;276: 364–377. doi: 10.1111/joim.12255 24717051 10.1111/joim.12255

[3] Saito M. Human brown adipose tissue: regulation and anti-obesity potential. Endocr J. 2014;61: 409–416. 24401694 10.1507/endocrj.ej13-0527

[4] Nedergaard J, Golozoubova V, Matthias A, Asadi A, Jacobsson A, Cannon B. UCP1: the only protein able to mediate adaptive non-shivering thermogenesis and metabolic inefficiency. Biochim Biophys Acta. 2001;1504: 82–106. 11239487 10.1016/s0005-2728(00)00247-4

[5] Seale P, Conroe HM, Estall J, Kajimura S, Frontini A, Ishibashi J, et al Prdm16 determines the thermogenic program of subcutaneous white adipose tissue in mice. J Clin Invest. 2011;121: 96–105. doi: 10.1172/JCI44271 21123942 10.1172/JCI44271 PMC3007155

[6] Nedergaard J, Cannon B. The browning of white adipose tissue: some burning issues. Cell Metab. 2014;20: 396–407. doi: 10.1016/j.cmet.2014.07.005 25127354 10.1016/j.cmet.2014.07.005

[7] Loyd C, Obici S. Brown fat fuel use and regulation of energy homeostasis. Curr Opin Clin Nutr Metab Care. 2014;17: 368–372. doi: 10.1097/MCO.0000000000000063 24839950 10.1097/MCO.0000000000000063

[8] Rosen ED, Spiegelman BM. What we talk about when we talk about fat. Cell. 2014;156: 20–44. doi: 10.1016/j.cell.2013.12.012 24439368 10.1016/j.cell.2013.12.012 PMC3934003

[9] Harms M, Seale P. Brown and beige fat: development, function and therapeutic potential. Nat Med. 2013;19: 1252–1263. doi: 10.1038/nm.3361 24100998 10.1038/nm.3361

[10] Stanton H, Rogerson FM, East CJ, Golub SB, Lawlor KE, Meeker CT, et al ADAMTS5 is the major aggrecanase in mouse cartilage in vivo and in vitro. Nature. 2005;434: 648–652. doi: 10.1038/nature03417 15800625 10.1038/nature03417

[11] Bauters D, Scroyen I, Deprez-Poulain R, Lijnen HR. ADAMTS5 promotes murine adipogenesis and visceral adipose tissue expansion. Thromb Haemost. 2016;116: 694–704. doi: 10.1160/TH16-01-0015 27383908 10.1160/TH16-01-0015

[12] Bauters D. C M, Geys L., Van Lint J., Hemmeryckx B., Lijnen H.R. Loss of ADAMTS5 enhances brown adipose tissue mass and promotes browning of white adipose tissue via CREB signaling. Molecular Metabolism. 2017. 10.1016/j.molmet.2017.05.004 PMC5485238 28702327

[13] Malfait AM, Ritchie J, Gil AS, Austin JS, Hartke J, Qin W, et al ADAMTS-5 deficient mice do not develop mechanical allodynia associated with osteoarthritis following medial meniscal destabilization. Osteoarthritis Cartilage. 2010;18: 572–580. doi: 10.1016/j.joca.2009.11.013 20036347 10.1016/j.joca.2009.11.013

[14] Bauters D, Spincemaille P, Geys L, Cassiman D, Vermeersch P, Bedossa P, et al ADAMTS5 deficiency protects against non-alcoholic steatohepatitis in obesity. Liver Int. 2016;36: 1848–1859. doi: 10.1111/liv.13181 27254774 10.1111/liv.13181

[15] McCulloch DR, Le Goff C, Bhatt S, Dixon LJ, Sandy JD, Apte SS. Adamts5, the gene encoding a proteoglycan-degrading metalloprotease, is expressed by specific cell lineages during mouse embryonic development and in adult tissues. Gene Expr Patterns. 2009;9: 314–323. doi: 10.1016/j.gep.2009.02.006 19250981 10.1016/j.gep.2009.02.006 PMC2725439

[16] Gorski DJ, Xiao W, Li J, Luo W, Lauer M, Kisiday J, et al Deletion of ADAMTS5 does not affect aggrecan or versican degradation but promotes glucose uptake and proteoglycan synthesis in murine adipose derived stromal cells. Matrix Biol. 2015;47: 66–84. doi: 10.1016/j.matbio.2015.03.008 25840345 10.1016/j.matbio.2015.03.008

[17] Ferrannini E. The theoretical bases of indirect calorimetry: a review. Metabolism. 1988;37: 287–301. 3278194 10.1016/0026-0495(88)90110-2

[18] Kleiner DE, Brunt EM, Van Natta M, Behling C, Contos MJ, Cummings OW, et al Design and validation of a histological scoring system for nonalcoholic fatty liver disease. Hepatology. 2005;41: 1313–1321. doi: 10.1002/hep.20701 15915461 10.1002/hep.20701

[19] Marchesi JR, Adams DH, Fava F, Hermes GD, Hirschfield GM, Hold G, et al The gut microbiota and host health: a new clinical frontier. Gut. 2016;65: 330–339. doi: 10.1136/gutjnl-2015-309990 26338727 10.1136/gutjnl-2015-309990 PMC4752653

[20] Tremaroli V, Backhed F. Functional interactions between the gut microbiota and host metabolism. Nature. 2012;489: 242–249. doi: 10.1038/nature11552 22972297 10.1038/nature11552

[21] Koza RA, Nikonova L, Hogan J, Rim JS, Mendoza T, Faulk C, et al Changes in gene expression foreshadow diet-induced obesity in genetically identical mice. PLoS Genet. 2006;2: e81 doi: 10.1371/journal.pgen.0020081 16733553 10.1371/journal.pgen.0020081 PMC1464831

[22] Bachmanov AA, Reed DR, Tordoff MG, Price RA, Beauchamp GK. Nutrient preference and diet-induced adiposity in C57BL/6ByJ and 129P3/J mice. Physiol Behav. 2001;72: 603–613. 11282146 10.1016/s0031-9384(01)00412-7 PMC3341942

[23] Collins S, Martin TL, Surwit RS, Robidoux J. Genetic vulnerability to diet-induced obesity in the C57BL/6J mouse: physiological and molecular characteristics. Physiol Behav. 2004;81: 243–248. doi: 10.1016/j.physbeh.2004.02.006 15159170 10.1016/j.physbeh.2004.02.006

[24] Lijnen HR. Reproducibility of studies with genetically modified mice. J Thromb Haemost. 2017;15: 1883–1884. doi: 10.1111/jth.13779 28696568 10.1111/jth.13779

[25] Westrick RJ, Ginsburg D. Modifier genes for disorders of thrombosis and hemostasis. J Thromb Haemost. 2009;7 Suppl 1: 132–135. 10.1111/j.1538-7836.2009.03362.x 19630785

[26] Kumar S, Sharghi-Namini S, Rao N, Ge R. ADAMTS5 functions as an anti-angiogenic and anti-tumorigenic protein independent of its proteoglycanase activity. Am J Pathol. 2012;181: 1056–1068. doi: 10.1016/j.ajpath.2012.05.022 22796434 10.1016/j.ajpath.2012.05.022

[27] Sharghi-Namini S, Fan H, Sulochana KN, Potturi P, Xiang W, Chong YS, et al The first but not the second thrombospondin type 1 repeat of ADAMTS5 functions as an angiogenesis inhibitor. Biochem Biophys Res Commun. 2008;371: 215–219. doi: 10.1016/j.bbrc.2008.04.047 18433719 10.1016/j.bbrc.2008.04.047

