# Supplementary figures and images for: Functional role of ADAMTS5 in adiposity and metabolic health
Source: PLoS One. 2018 Jan 2;13(1):e0190595. doi: 10.1371/journal.pone.0190595 (PMC5749841; doi:10.1371/journal.pone.0190595)

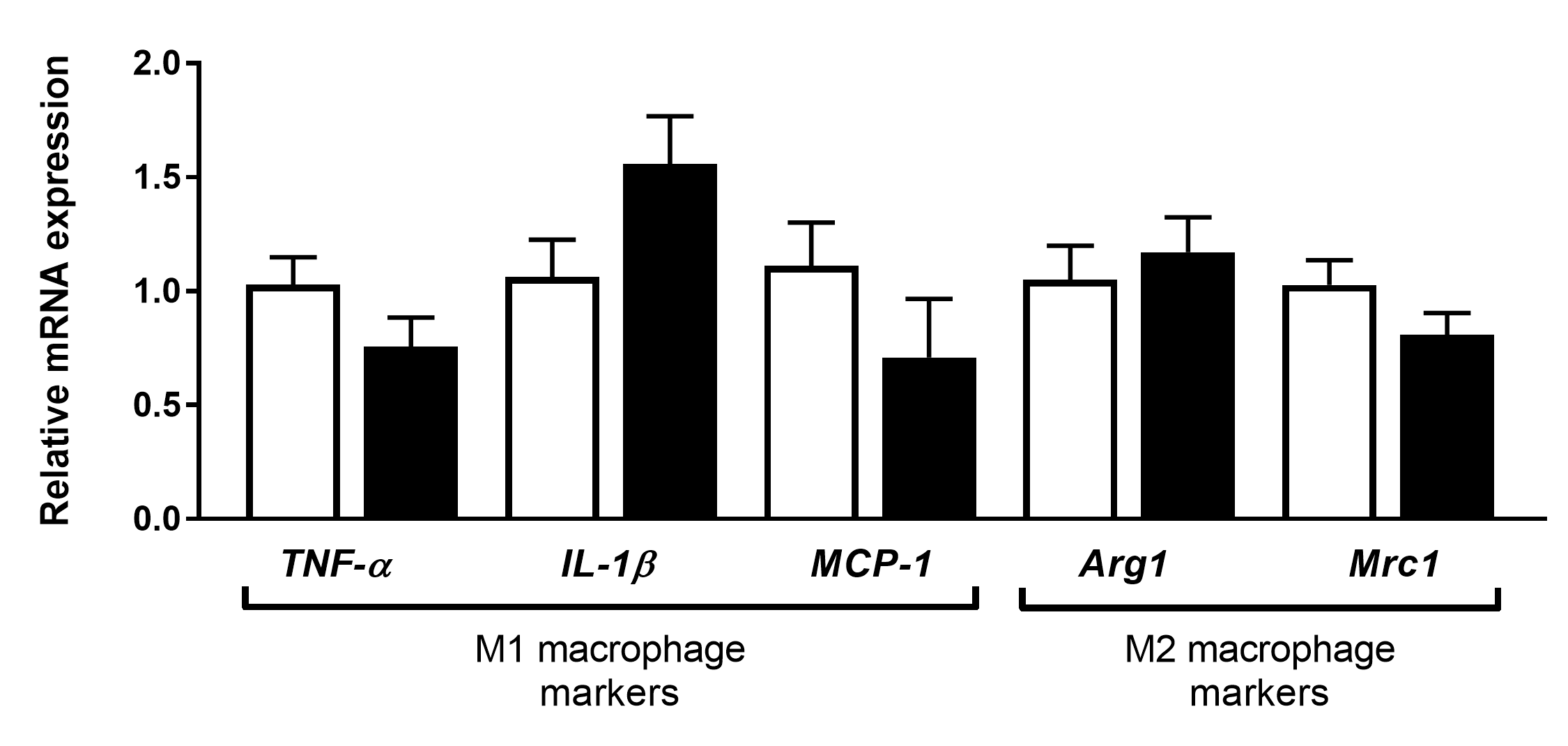

Supplement: S1 Fig — Gene expression levels of M1 macrophage markers (Tnf-α (Tumour necrosis factor-α); Il-1β (Interleukin-1β) and Mcp-1 (Monocyte chemoattractant protein-1)) and M2 macrophage markers (Arg1 (Arginase 1) and Mrc1 (Mannose receptor c type 1)) in livers of obese WT (white bars) and Adamts5-/--J (black bars) mice were normalized to the housekeeping gene β-actin and shown relative to WT. Data are means ± SEM of 6 (WT) or 7 (Adamts5-/--J) experiments. ADAMTS5, a disintegrin and metalloproteinase with thrombospondin type 1 motifs member 5; WT, wild-type; HFD, high-fat diet. (TIF) [file pone.0190595.s001.tif]

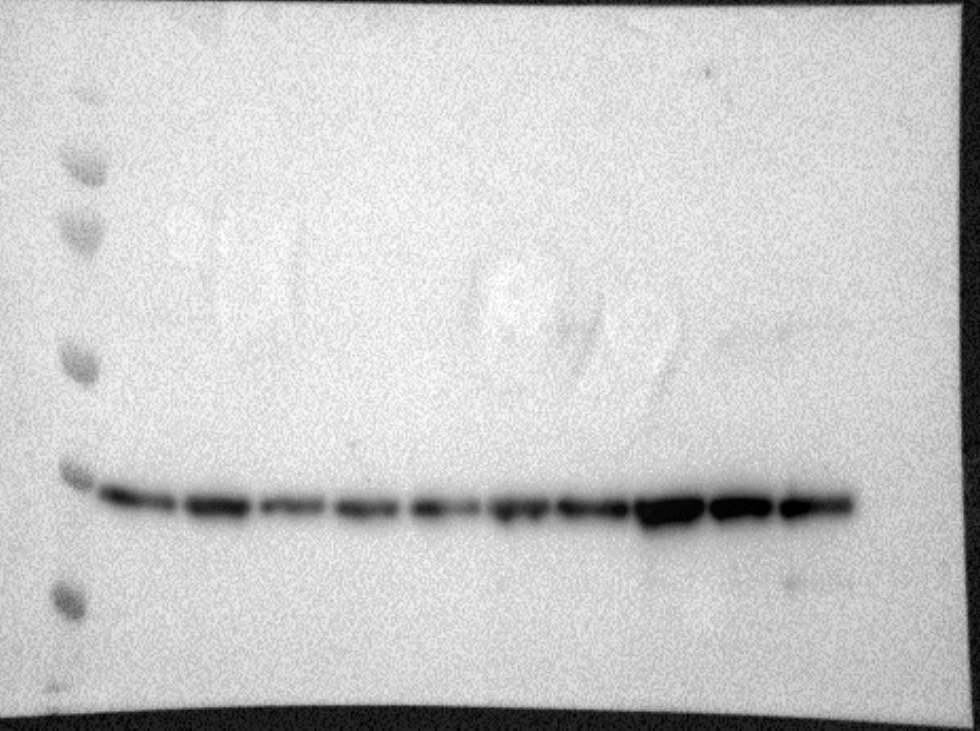

Supplement: S1 Data — (ZIP) [file pone.0190595.s002.zip › Fig1.tif]

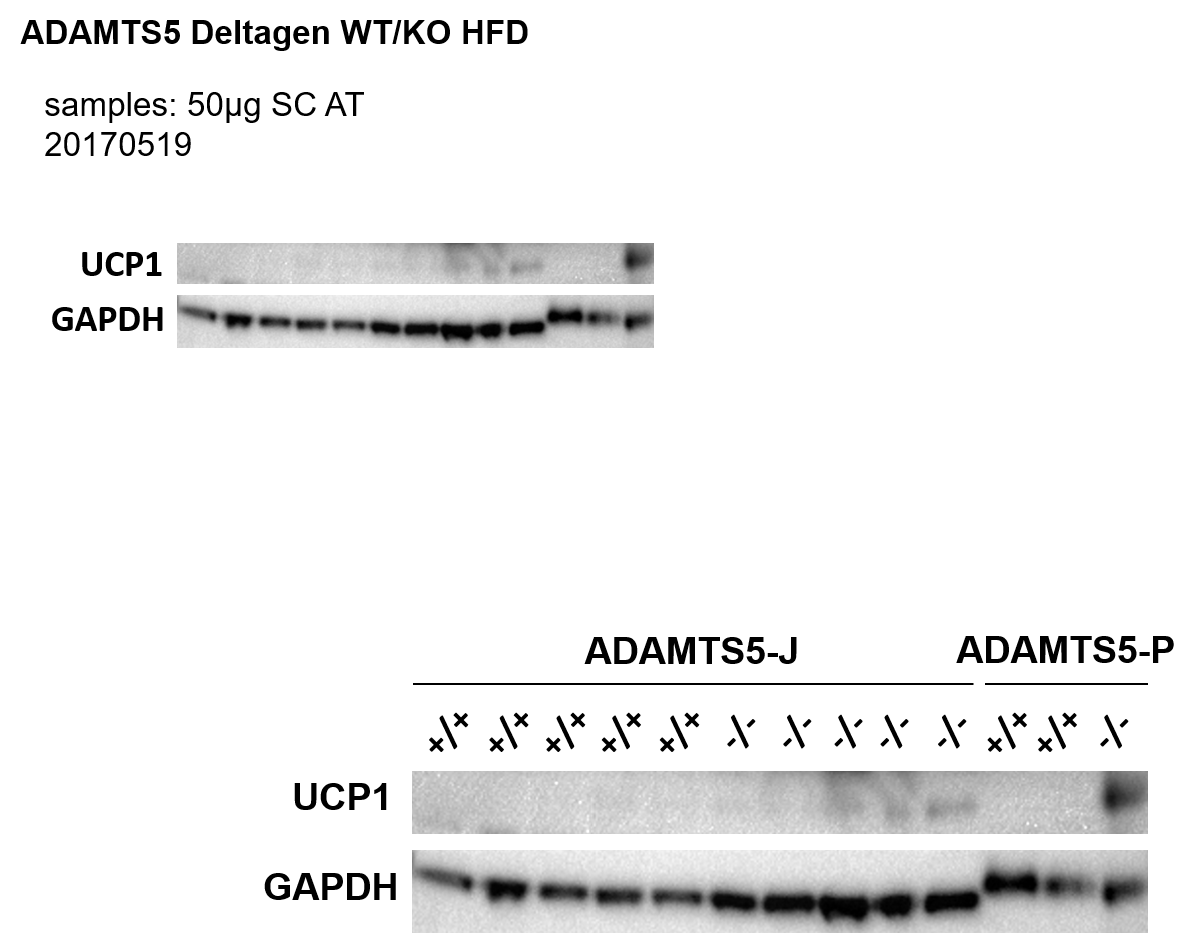

Supplement: S1 Data — (ZIP) [file pone.0190595.s002.zip › Fig2 panel D UCP1 protein levels ADAMTS5_J and ADAMTS5_P.tif]

## Slide 1
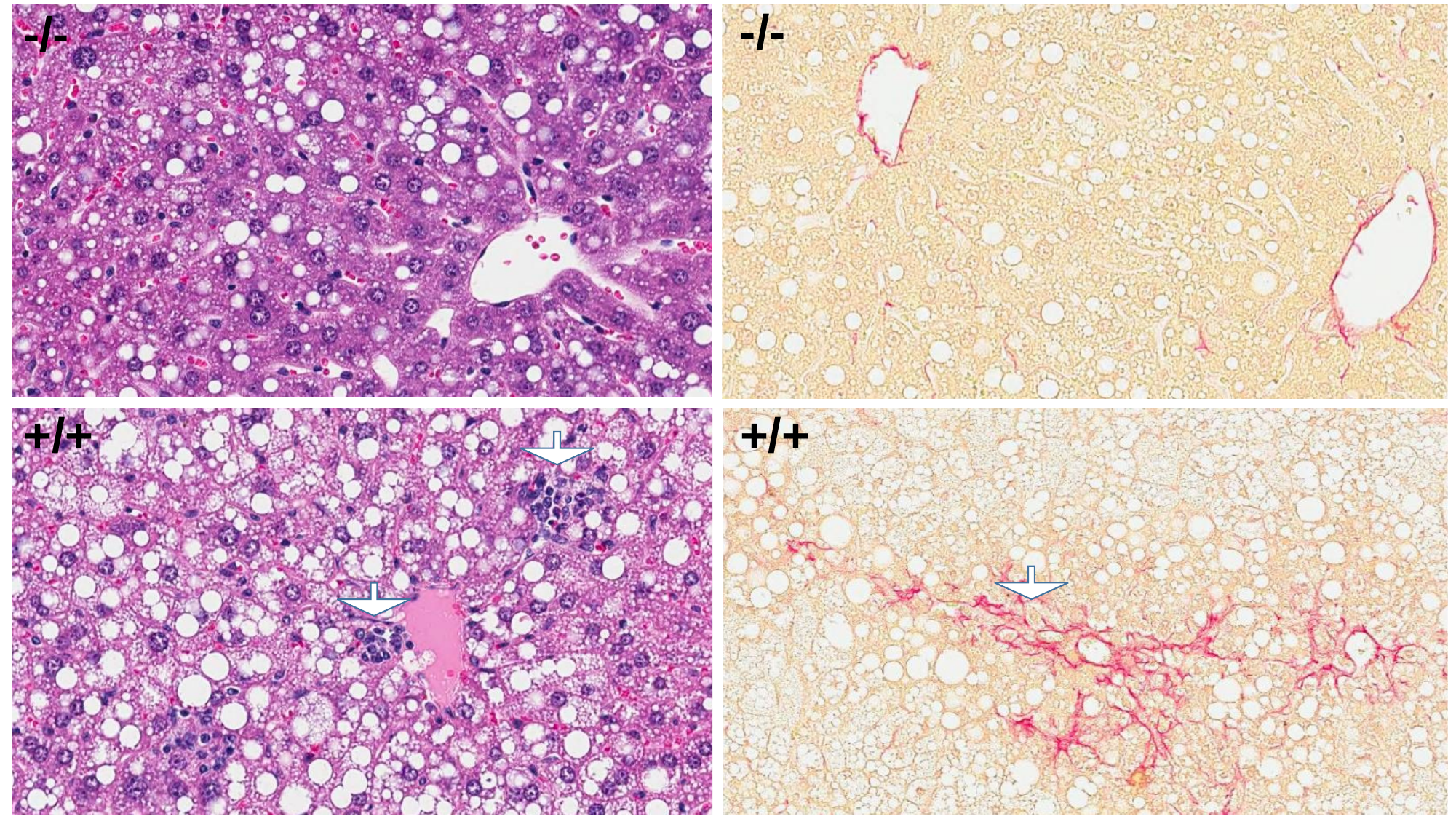

-/-
-/-
+/+
+/+

Supplement: S1 Data — (ZIP) [file pone.0190595.s002.zip › Fig7 panel A.pptx]
